# Atherogenic Dyslipidemia in Children: Evaluation of Clinical, Biochemical and Genetic Aspects

**DOI:** 10.1371/journal.pone.0120099

**Published:** 2015-04-21

**Authors:** Anna Montali, Gessica Truglio, Francesco Martino, Fabrizio Ceci, Giampiero Ferraguti, Ester Ciociola, Marianna Maranghi, Francesco Gianfagna, Licia Iacoviello, Roberto Strom, Marco Lucarelli, Marcello Arca

**Affiliations:** 1 Department of Internal Medicine and Allied Sciences, Atherosclerosis Unit, Sapienza University of Rome, Rome, Italy; 2 Department of Cellular Biotechnologies and Hematology, Sapienza University of Rome, Rome, Italy; 3 Department of Pediatrics, Sapienza University of Rome, Rome, Italy; 4 Centro Ricerche Epidemiologia e Medicina Preventiva, Università dell'Insubria, Varese, Italy; 5 Department of Epidemiology and Prevention, Laboratory of Molecular and Nutritional Epidemiology, Istituto di Ricovero e Cura a Carattere Scientifico, Istituto Neurologico Mediterraneo, Pozzilli, Italy; 6 Pasteur Institute—Cenci Bolognetti Foundation, Sapienza University of Rome, Rome, Italy; Wake Forest School of Medicine, UNITED STATES

## Abstract

The precursors of atherogenic dyslipidemia (AD) are not well defined. Therefore, we investigated 62 non-obese, non-diabetic AD and 221 normolipemic children. Anthropometric parameters, blood pressure and biochemical measures were obtained in index children, their parents and all available siblings. The heritability (h^2^) of anthropometric and biochemical traits was estimated by SOLAR. Rare and common variants in *APOA1* and *LPL* genes were screened by re-sequencing. Compared to normolipemic, AD children showed increased body mass index, waist circumference, plasma glucose, insulin, ApoB, HOMA-IR, hs-CRP and lower adiponectin (p<0.001 for all). Metabolic syndrome was present in 40% of AD while absent in controls. All traits (except adiponectin and hs-CRP) showed a strong familial aggregation, with plasma glucose having the highest heritability (89%). Overall, 4 *LPL *loss-of-function mutations were detected (p.Asp9Asn, p.Ser45Asn, p.Asn291Ser, p.Leu365Val) and their cumulative prevalence was higher in AD than in control children (0.073 *vs*. 0.026; P=0.038). The *LPL *p.S447* gain-of-function mutation, resulted to be less frequent in AD than in control children (0.064 *vs*. 0.126; P=0.082). No variant in the *APOA1 *gene was found. Our data indicate that AD is a rather common dyslipidemia in childhood; it associates with metabolic abnormalities typical of insulin resistant state and shows a strong familial aggregation. *LPL *variants may contribute to the development of AD phenotype.

## Introduction

Atherogenic dyslipidemia (AD) is a highly atherogenic lipid abnormality characterized by the combination of increased plasma concentration of triglycerides (TG), reduced high-density lipoprotein cholesterol (HDL-C) and increased numbers of small, dense low-density lipoprotein (sdLDL) particles [1, 2]. AD is thought to occur mainly in adulthood due to a combination of genetic predisposition with aging and adiposity [3, 4]. Nevertheless, epidemiological observations have reported that the lipid components of AD—namely low HDL-C and high TG—may be present since childhood [5], thus providing the rationale for searching this form of dyslipidemia also at younger ages. The earlier identification of AD is justified by the fact that these lipid abnormalities in children and adolescents have been demonstrated to predict clinical manifestation of atherosclerosis in adulthood [6].

Conditions associated to AD are central obesity and insulin resistance, which are, in turn, strongly associated with a low-grade inflammation state and abnormalities in adipokines production [7]. Indeed, in adults with AD higher levels of serum insulin and C reactive protein (CRP) and lower levels of adiponectin have been also reported [8]. However, if these abnormalities are early or late biochemical markers of AD phenotype is not known. The investigation of a cohort of children could help in shedding light into this question.

Although it is well recognized that also genetic factors predispose to the development of AD, few studies have investigated the familial aggregation of components of this lipid phenotype. The only indirect estimates derives from studies in metabolic syndrome (MetS) whose components (insulin resistance, obesity, hypertension, and serum lipids) showed heritability values ranging from 20% to 80% [9]. On the other hand, a number of candidate genes have been linked to the lipoprotein abnormalities of AD. Among them, genes coding for lipoprotein lipase (*LPL*) and apolipoprotein AI (*APOAI*) appear to be particularly relevant due to their pivotal role in regulating both TG and HDL-C levels [10–12]. Though variants in these genes may predispose to AD, direct data in children are scanty.

Therefore, within a large investigation dedicated at dyslipidaemia in the youth, we designed this study aimed at describing the metabolic and genetic abnormalities associated to AD in children. As lipid abnormalities cluster within families, an additional aim of this study was to evaluate the familial aggregation of traits associated with this disorder and to estimate the contribution of rare and common genetic variants into *LPL* and *APOA1* genes to the lipid components of AD.

## Material and Methods

### Subjects’ selection and classification

For this study we have considered all children (2–18 years) referred between November 2006 and January 2012 to the paediatric Lipid Clinic of our University Hospital for suspected dyslipidaemia [13]. At first visit, anthropometric (body weight, waist, hip and arm circumferences) and lipoprotein measurements were obtained in index children, their parents and all available siblings. In children, weight was measured using an electronic scale (Soehnle, Murrhardt, Germany), and the standing height was measured with the Harpenden Stadiometer (Holtain, Crymych, Great Britain). Systolic and diastolic blood pressure was measured by using a random zero sphygmomanometer (Hawksley &Sons Ltd, Lancing, UK); the mean of three measurements was used in the analysis. Body mass index (BMI) was calculated as weight/ height^2^ (kg/m^2^).

Children showing plasma levels of TG ≥90^th^ and HDL-C ≤10^th^ age and sex-specific percentiles were classified as AD [14], while those with TG<75^th^ and HDL>10^th^ were used as comparator. TG and HDL-C percentiles reported for Italian children and adult (>20 years) populations were used as reference values [15, 16]. Children with clinical diagnosis of dominant familial hypercholesterolemia (ADH) were excluded. The criteria for diagnosing ADH have been previously reported [13]. Additional exclusion criteria were thyroid dysfunction, diabetes mellitus, nephrotic syndrome and liver disease as detected by standard laboratory tests. Subjects taking statins or other medications for lipoprotein disorder were also excluded from the analyses.

The presence of MetS in adults was defined according to the American Heart Association (AHA) criteria, [17]. To define MetS in children, we used the pediatric AHA definition which is based upon the AHA adult definition but uses pediatric reference standards for blood pressure (BP), waist circumference (WC), TGs and HDL-C [18, 19]. Thus, in our study central obesity was defined as a WC ≥ 90th percentile for age and gender; hyperTG as triglycerides ≥ 90th percentile for age and gender; low HDL-C as concentrations ≤10th percentile for age and gender; elevated BP as systolic or diastolic BP ≥90th percentile for age, gender, and height percentile; and impaired fasting glucose as glucose ≥ 100 mg/dl.

Written informed consent on behalf of enrolled children was obtained from parents and all adults gave their written consent before entering into the study. The study was approved by the Institutional Review Board and carried out in accordance with the Helsinki Declaration.

### Biochemical measurements

Blood samples were collected early in the morning after an overnight fast in EDTA-containing tubes. Plasma concentrations of lipoprotein apoproteins, blood glucose and insulin were determined as previously described [13, 20]. Insulin resistance was estimated by HOMA_IR_ as previously described [21]. Plasma levels of high molecular weight (HMW) adiponectin levels were determined by ELISA kit (EZHMWA A-64K, Millipore, Missouri, U.S.A) according to manufacturer’s instructions. Plasma high-sensitive C-reactive protein (hs-CRP) concentrations were analyzed by particle-enhanced turbidimetric immunoassay (C-Reactive Protein Latex High Sensitivity—Roche Diagnostics GmbH, D-68298 Mannheim) following manifacturer’s instructions.

### Genetic analysis

Genomic DNA of 55 AD and 174 normolipemic children was obtained from peripheral blood leukocytes by either the salting-out method [22] or the QIAamp 96 Blood DNA kit (Qiagen, Hilden, Germany). The mutational search in *LPL* and *APOA1* genes was performed using the robotic system Microlab Starlet (Hamilton, Reno, USA) for the preparation of the reactions in a 96-well and the ABI PRISM 3130*xl* (Applied Biosystems, Foster City, USA) genetic analyzer for targeted sequencing. For the purpose of confirmation, the SNPs identified in the *LPL* gene were also investigated by using the SNaPshot assay (Life Technologies, Carlsbad, USA). Details of procedures are reported in the S1 File, S1 Table and S2 Table.

### Statistical analysis

Statistical analyses were performed using the SPSS package (version 18.0; SPSS Inc., Chicago, IL, USA) and STATA v.11. The data are expressed as either frequencies or means with standard deviation. The variables showing a skewed distribution were reported as median (interquartile ranges) and tested after log transformation. Student’s *t*-test for unpaired data or U Mann-Whitney were used to compare continuous variables, while either Fisher’s exact test or **χ** ^**2**^ test was used to compare categorical variables. All comparisons were adjusted for age, sex and BMI by using the generalized linear model (GLM). Pearson’s correlation and linear regression coefficients were used to examine the relationship between variables.

The computer package SOLAR (Sequential Oligogenic Linkage Analysis Routines, version 4.2.7) was used for heritability analyses [23]. A maximum likelihood-based variance decomposition approach was used to estimate the proportion of the overall variability due to genetic (heritability, h^2^) and covariates effects. The significance was assessed by means of the likelihood ratio test, using the tdist command when residual kurtosis of the traits was >0.8, allowing a robust estimation of mean and variance of the trait. Covariates used in models included age, sex, age*sex (sex modality was 1 or 2), entering in the final model if *p*<0.1. The analysis was repeated after stratification for case and control families, maintaining statistical models and settings used for the related analysis in the whole sample. Before analysis, the distribution of variables was assessed using Shapiro-Wilk tests and variables not normally distributed were transformed when appropriate using the statistical package SAS (version 9.1.3 for Windows, Cary, NC: SAS Institute Inc.).

We also assessed the odds ratios for the presence of AD in the parents according to the status of the proband using a logistic regression model, with and without adjustment for age, age square, gender and BMI. The general estimated equation model was used to account for the correlation among siblings within a family. In addition, the probability (odds) of a child with AD to having a parent with the same lipid phenotype has been estimated by using logistic regression analysis where age, sex and BMI were included as covariates.

All statistical tests were 2-sided and a P <0.05 was set as the statistical significance level.

## Results

### Identification of AD children and families

**Fig 1:**
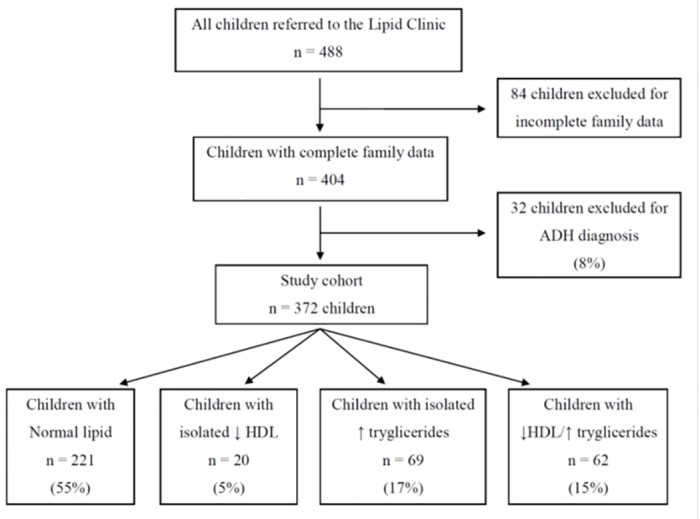
Flow diagram for classification of children with atherogenic dyslipidemia.

A total number of 488 Caucasian index children were originally considered for the study. Fig 1 shows the flow diagram for classification of these children. Eighty four (17.2%) were excluded due to incomplete family data, thus leaving a study cohort of 404 children. According to the predefined criteria, 221 children (54.7%) (89 boys and 132 girls) were classified as without AD, 32 (7.9%) with autosomal dominant hypercholesterolemia (ADH) and 89 (21.9%) with either isolated low HDL-C or isolated high TG. Therefore, 62 (41 boys and 21 girls) were classified as affected by AD providing a prevalence of 15.3%. Within families with AD we examined 124 parents (62 father and 62 mothers) and 43 siblings (22 brothers and 21 sisters); within families with normolipemic children we examined 442 parents (221 father and 221 mothers) and 160 siblings (91 brothers and 69 sisters).

### Comparison of clinical and biochemical characteristics of children with and without AD

Table 1 shows the comparison of clinical and biochemical characteristics between children with AD and those with normal lipids. Age and gender were not significantly different between groups, but male sex appeared to be more frequent among AD children. Although LDL-C and apoB, levels did not differ between groups, the LDL-C/Apo B ratio was lower in AD compared to controls children. As reduced LDL-C/apoB has been reported to be a good predictor of smaller LDL particles [24], this finding strongly suggests an increased prevalence of sdLDL in the AD group. Respect to controls, BMI and waist circumference were significantly greater in children with AD and these difference was present in both boys and girls (data not shown). The differences in the waist circumference remained statistically significant (p = 0.038) also when adjusted for age and sex. Plasma levels of fasting blood glucose, insulin, HOMA-IR and hs-CRP were higher and those of HMW adiponectin were lower in children with AD compared to controls. With the exception of those in adiponectin levels, all these differences remained statistically significant after adjustment for age, waist circumference and gender. Conversely, systolic and diastolic blood pressure did not differ significantly between groups. Twenty-six children with AD (42%) were found to have MetS compared to none among controls.

**Table 1:** Clinical characteristics and laboratory parameters of children with and without atherogenic dyslipidemia.

Variables	Children with atherogenic dyslipidemia (n = 62)	Children without atherogenic dyslipidemia (n = 221)
**Age**, (yrs)	8.2 ± 3.9	8.3 ± 3.4
**Sex, Boys**	41 (66%)	89 (40%)
** Girls**	21 (34%)	132 (60%)
**BMI**, (kg/m^2^)	21.1 ± 5.3***	18.2 ± 3.5
**Systolic BP** (mmHg)	106.8 ± 10.7	108.1 ± 11.2
**Diastolic BP** (mmHg)	68.3 ± 10.0	70.3 ± 8.9
**Waist circumference** (cm)	73.0 ± 16.8***	61.5 ± 9.6
**Plasma lipids** (mg/dl)		
** Total cholesterol**	187.3 ± 37.4	198.6 ± 38.7
** HDL-C**	32.9 ± 5.0***	58.8 ± 12.1
** Non-HDL**-C	154.4 ± 36.2***	139.8 ± 38.3
** Total triglycerides**	158.9 ± 83.2***	65.0 ± 19.2
** LDL-C**	122.5 ± 38.1	126.9 ± 37.4
** ApoB**	89.6 ± 27.5	82.4 ± 22.4
** LDL-C/Apo B**	1.39 ± 0.32**	1.65 ± 0.75
** ApoAI**	126.0 ± 25.6***	155.3 ± 30.9
**Fasting Blood Glucose** (mg/dl)	77.2 ± 11.3***	69.1 ± 11.0
**Insulin** (μU/ml)	11.03 ± 8.36***	6.46 ± 4.23
**HOMA-IR**	2.14 ± 1.64***	1.09 ± 0.8
**hs-CRP** (mg/dl)	0.08 (0.04–0.22) *	0.03 (0.00–0.12)
**Adiponectin** (μg/ml)	3.75 (2.45–6.04) ***	5.67 (3.88–7.41)
**Metabolic Syndrome**, n (%)	26 (42) ***	0

BMI, body mass index; BP, blood pressure; HDL-C, high density lipoprotein cholesterol; LDL-C, low density lipoprotein cholesterol; ApoB, apolipoprotein B; ApoAI, apolipoprotein AI; hs-CRP, high sensitivity C Reactive Protein; HOMA-IR, homeostasis model assessment of IR index.

hs-CRP and adiponectin values are reported as median (interquartile range)

* p<0.05;

** p<0.01;

*** p<0.001

Univariate correlations between clinical and biochemical characteristics in the whole group of study children are reported in S3 Table. Age was a significant determinant of FBG, insulin, HOMA-IR, BMI and waist circumference (positive) and adiponectin (negative). As expected, BMI and waist circumference were significantly correlated with indices of insulin resistance (positively) and TG and with adiponectin, HDL-C (negatively). Finally, hs-CRP showed a slight, positive correlation with plasma insulin and HOMA and negative with HDL-C and ApoAI.

### Familial aggregation of AD traits

To evaluate the familial aggregation of the AD phenotype, we first compared plasma lipids and other characteristics of parents and siblings of children with and without AD (Table 2). Parents of AD children showed significantly higher non HDL-C, TG, LDL-C, apoB and lower HDL-C and ApoAI levels compared to those of children without AD. Also, mean levels of fasting blood glucose and insulin levels were higher in parents of children with AD compared to controls, even though only the former reached a level of statistical significance. This translated in higher HOMA index in parents of children with AD, but the difference did not reach the statistical significance. Even though BMI was comparable between groups, the waist circumference was significantly greater in parents of children with dyslipidemia compared with controls indicating a tendency towards increased prevalence of visceral obesity among this group. Finally, when we compared the prevalence of MetS between groups, it was found to be almost 3 times more frequent among parents of dyslipidemic children and this difference was highly significant (p<0.001).

**Table 2:** Clinical characteristics and laboratory parameters of parents and siblings of children with and without atherogenic dyslipidemia.

	Parents		Siblings	
Variables	of children with atherogenic dyslipidemia(n = 124)	of children without atherogenic dyslipidemia (n = 442)	of children with atherogenic dyslipidemia (n = 43)	of children without atherogenic dyslipidemia (n = 160)
**Age**, (yrs)	40.0 ± 6.4	40.4 ± 5.8	11.2 ± 5.8	10.2 ± 6.2
**BMI**, (kg/m^2^)	25.5 ± 3.9	24.9 ± 4.0	21.0 ± 6.2	18.7 ± 3.9
**Systolic BP** (mmHg)	119.8 ± 13.0	119.2 ± 13.2	102.3 ± 12.2	104.7 ± 12.3
**Diastolic BP** (mmHg)	75.7 ± 8.5	76.4 ± 9.3	63.4 ± 7.2	66.4 ± 8.4
**Waist circumference** (cm)	93.0 ± 13.0*	88.9 ± 13.3	74.9 ± 25.1	60.3 ± 12.8
**Plasma lipids** (mg/dl)				
** Total cholesterol**	211.8 ± 46.2	205.2 ± 38.7	166.9 ± 35.9	171.1 ± 38.46
** HDL-C**	45.0 ± 12.3***	54.2 ± 14.5	42.4 ± 9.0^**‡‡‡**^	55.0 ± 12.4
** Non HDL-C**	166.8 ± 47.6***	150.9 ± 41.8	124.4 ± 37.1	116.1 ± 36.0
** Total triglycerides**	141.5 ± 88.6***	103.7 ± 83.3	98.7 ± 63.2^**‡‡‡**^	63.7 ± 23.5
** LDL-C**	139.0 ± 44.3*	130.1 ± 34.5	104.6 ± 34.1	103.3 ± 34.8
** ApoB**	94.4 ± 30.1**	86.7 ± 25.6	74.5 ± 24.9	73.5 ± 22.8
** LDL-C/ApoB**	1.51 ± 0.41	1.60 ± 0.56	1.49 ± 0.44	1.51 ± 0.67
** ApoAI**	147.8 ± 32.0***	159.1 ± 34.6	133.9 ± 27.1^**‡‡‡**^	154.1 ± 31.0
**Fasting Blood glucose** (mg/dl)	83.2 ± 19.7***	75.9 ± 13.3	77.7 ± 12.8	72.0 ± 11.4
**Insulin** (μU/ml)	9.68 ± 14.0	7.93 ± 4.80	8.5 ± 7.2	6.95 ± 5.35
**HOMA-IR**	2.54 ± 7.31	1.55 ± 1.28	1.81 ± 1.97	1.30 ± 1.07
**hs-CRP** (mg/dl)	0.11 (0.04–0.29)	0.09 (0.03–0.22)	0.04 (0.01–0.11)	0.05 (0.01–0.14)
**Adiponectin** (μg/ml)	3.44 (2.22–4.88)	3.20 (2.09–5.46)	—-	—-
**Metabolic Syndrome, n (%)**	9 (7.2) ***	12 (2.7)	5 (11.6) ^**‡‡‡**^	—-

BMI, body mass index; BP, blood pressure; HDL-C, high density lipoprotein cholesterol; LDL-C, low density lipoprotein cholesterol; ApoB, apolipoprotein B; ApoAI, apolipoprotein AI; hs-CRP, high sensitivity C Reactive Protein; HOMA-IR, homeostasis model assessment of IR index. hs-CRP and adiponectin values are reported as median (interquartile range)

* P<0.05;

** p<0.01;

*** p<0.001 between parents

^**‡‡**^ p<0.01;

^**‡‡‡**^ p<0.001 between siblings

The comparison of demographic and metabolic parameters between siblings of children with and without AD showed somewhat similar differences (Table 2). In fact, brothers and sisters of affected children showed, on average, significantly higher TG and lower HDL-C and apoA1 respect to those of normolipemic children; they also presented a tendency towards higher indices of insulin resistance and visceral obesity, but the differences were not statistically significant. Nonetheless, among siblings of AD children we observed a markedly increased prevalence of MetS. To explore the extent to which AD aggregates within families further, we estimated the heritability (h^2^) of the phenotypic traits in the study groups (S4 Table). All traits, except adiponectin and hs-CRP, showed a significant genetic component, with glucose levels having the highest heritability. Plasma levels of lipoproteins as well as apolipoprotein and their ratios also showed strong genetic influence presenting h^2^ values ranging from 52% to 83%. The groups of children with AD showed higher heritability levels for anthropometric measures and non-HDL-C.

### Genetic analysis of *LPL* and *APOA1* genes in children with and without AD

The analysis of *APOA1* gene did not reveal any variants in both groups of children. Conversely, the screening of *LPL* led to identification of 8 exonic variants, 3 of which were synonymous. Of the 5 non-synonymous variants, 4 were missense and 1 was nonsense (Table 3). Most of them (p.Asp9Asn, p.Asn291Ser, p.Leu365Val and p.S447*) have been previously reported [25–27], while the p.Ser45Asn variant is described here for the first time: All variants were found to be at heterozygous state. The child carrying the p.Asp9Asn resulted to be heterozygous for the complex allele [c.-281T>G; c.106G>A] with the two variants in *cis* on the same allele. The segregation analysis in parents assigned this complex allele to the father. The novel *LPL* c.215 G>A (p.Ser45Asn) variation was identified in a child showing TGs of 257 and HDL-C of 30 mg /dl. It was inherited from the father who also showed TGs 232 and HDL-C 39 mg/dl. It is located in the amino-terminal domain of the protein that represents the site with the catalytic activity and it is also close to a glycosylation site critical for the proper maturation of the protein. Furthermore, the *in silico* analysis indicated that it could be probably damaging (PolyPhen-2 score = 0.099; Panther, P_deleterious_ = 0.221). Taking together, these results highlight that p.Ser45Asn might have a functional effect.

**Table 3:** Prevalence in the cohort of probands with atherogenic dyslipidemia of non-synonymous exonic variants in the LPL gene.

Position	Variant name			Allele frequencies			Comparison in respect to controls without AD			Comparison in respect to other databases		
	DNA level	Protein level^a^	SNP identifier	Probands with AD	Controls without AD	Other databases	P value	Odds ratio	95% confidence interval	P value	Odds ratio	95% confidence interval
Exon 2	c.106 G>A	p.Asp9Asn	rs1801177	A = 0.009 (n = 1/110)	A = 0.020 (n = 7/348)	A = 0.013^**b**^ (n = 10/758)	P = 0.686^**d**^	0.447	0.054 to 3.675	P = 1.000^**d**^	0.686	0.087 to 5.416
Exon 2	c.215 G>A	p.Ser45Asn	new^#^	A = 0.009 (n = 1/110)	A = 0 (n = 0/348)	—	P = 0.240^**d**^	9.548	0.386 to 236.3	—	—	—
Exon 6	c.953 A>G	p.Asn291Ser	rs268	G = 0.045 (n = 5/110)	G = 0.006 (n = 2/348)	G = 0.016^**b**^ (n = 12/758)	*P = 0.010^**d**^	8.238	1.575 to 43.10	P = 0.053^**d**^	2.960	1.022 to 8.573
Exon 8	c.1174 C>G	p.Leu365Val	rs118204078	G = 0.009 (n = 1/110)	G = 0 (n = 0/348)	G = 0.0001^**c**^ (n = 1/8600)	P = 0.240^**d**^	9.548	0.386 to 236.3	*P < 0.0001^**e**^	78.89	4.899 to 1270
Exon 9	c.1421 C>G	p.S447*	rs328	G = 0.064 (n = 7/110)	G = 0.126 (n = 44/348)	G = 0.123^**b**^ (n = 93/758)	P = 0.082^**d**^	0.470	0.205 to 1.075	P = 0.078^**d**^	0.486	0.219 to 1.077

^**a**^The name at protein level is without the pro-peptide.

n = number of variant alleles / number of total alleles

^**b**^Frequency from database 1000 genomes EUR;

^**c**^Frequency from ESP6500:European_American, the only available for L365V SNP.

Depending on the sample size and absolute frequencies in the contingency table:

^**d**^Fisher exact test or

^**e**^χ^2^ was applied.

^#^ = annotations for the new variant are: NC_000008.11:g.19948306G>A; NG_008855.1:g.14236G>A; NM_000237.2:c.215G>A; NP_000228.1:p.Ser45Asn

* = statistically significant at indicated P level.

When the individual frequencies of *LPL* variants were compared between groups, we noticed that the p.N291S variant allele was significantly more common in AD than in control children (0.045 *vs*. 0.006; p = 0.010) while, at the opposite, the p.S447* allele tended to be more prevalent in controls than in AD children (respectively 0.126 *vs*. 0.064, p = 0.082). The difference in the prevalence of the other *LPL* variant alleles did not reach the statistical significance.

As the p.Asp9Asn, p.Asn291Ser and p.Leu365Val variants have been already demonstrated causing loss-of-function (LOF) alleles [28] and the novel p.Ser45Asn was classified as probably LOF, their cumulative prevalence was compared between AD and controls. We found that *LPL* LOF alleles were significantly more frequent in AD than in control children (0.073 *vs*. 0.026, respectively; p = 0.038) determining for AD children an almost 3 times higher risk of being carriers of any of them (HR = 2.95; 95% CI 1.11–7.86; p = 0.038). As the p.S447* is a gain-of-function *LPL* variant associated with low TG levels [29], we performed a further analysis classifying *LPL* alleles in 3 classes (with the p.S447X, with all LOF and with wild type). A statistically significant difference of their distribution between AD and control children was evidenced (χ^2^ = 7.931, p = 0.019).

The comparison of lipid profile between AD children with (n = 8) and without (n = 40) LOF alleles in the *LPL* demonstrated that carriers had higher TG levels compared to non-carriers (175 ± 96 mg/dl *vs*. 153 ± 86 mg/dl, respectively), even though the difference was not statistically significant probably due to the small sample size.

## Discussion

In this study, we sought to evaluate the metabolic and the genetic features of paediatric AD. Within a large cohort of children referred to a tertiary paediatric lipid clinic we found that about 15% of them showed a lipid phenotype classifiable as AD. Although this figure cannot be considered representative of the prevalence of AD in the general population of children, it suggests that AD is a rather frequent lipid abnormality among children with dyslipidaemia, certainly more common than dominant hypercholesterolemia (7.9%) and just lower than that of isolated hypertriglyceridemia (17.0%). We have classified AD based upon arbitrary age- and sex- specific lipoproteins cut-off values, and some have raised concern about the reliability of this criterion for classifying dyslipidemia in children [30]. Nevertheless, we found that AD children showed a constellation of several metabolic abnormalities such as greater waist circumference, higher fasting levels of plasma glucose and insulin and reduced insulin sensitivity, higher levels of his-CRP and lower levels of adiponectin. It is also noteworthy that, compared to controls, AD children showed an abnormal composition of LDL fraction as suggested by the increased LDL-C/ApoB ratio. All these abnormalities have been already reported in adults diagnosed with AD [3, 8], thus suggesting that they may represent early events in the development of the AD trait. In addition, this finding indicates that the AD classification we used identified a group of children showing an array of generalized metabolic disorders.

It has been reported in adulthood that AD is a component of MetS [31]. This seems to be partially true in childhood as we observed that only about 40% of AD children showed features of MetS. This latter finding is consistent with previous observations indicating that at younger ages (<35 years) AD is a less robust identifier of MetS, but may reflect the predisposition to an isolated lipid disorder [32]. It must be, however, recognized that we cannot exclude that AD in many children might foreshadows the development of MetS later in life. Moreover, AD has been reported to be strongly related with obesity [4]. However, it must be noted that the multiple metabolic abnormalities in AD children, although associated with increased waist circumference, developed in the context of borderline-normal body fat and persisted even after adjustment for measures of adiposity. One possible interpretation of our data is that the underlying metabolic defects in AD children reflect an insulin-resistant state that extends beyond adipose tissue. Indeed, previous investigators have postulated a condition in which a primary condition of insulin resistance is responsible for multiple metabolic disorders, including dyslipidemia [33]. In addition, the observation that parents and siblings of AD children showed predominantly abnormal lipid concentrations (and to a lesser extent visceral obesity and insulin resistance) might also argue in favour for the co-existence of inherited defects in apoB-containing lipoproteins metabolism in AD [34]. Indeed, it would be not surprising that an insulin resistant state on a background of genetic susceptibility to metabolic abnormalities worsens the expression of dyslipidemia.

An important finding of our study was that several traits associated to AD showed high degree of heritability. Our results are in line with previous studies that provided evidence for a strong association between parental metabolic disorders and abdominal obesity, high triglycerides and low HDL-C in their offspring [35]. Interestingly, in our AD families, beside plasma glucose, non-HDL-C and apoB presented the highest levels of heritability thus arguing in favor for the existence of an inherited defect in apoB-containing lipoproteins metabolism in AD children. It must be noted, however, that our children shared the same family environment or behaviour and this may emphasis the role of inheritance. Nonetheless, our data strongly argue in favour of a significant genetic component in AD.

In this study, we have also investigated the role of *LPL* and *APOAI* as candidate genes in predisposing children to develop AD. No sequence changes were found in *APOAI*, but 14.5% of AD probands presented variants in *LPL* gene. AD children as a group showed 3 times higher frequency of heterozygous LOF mutations in this gene. Moreover, the presence of *LPL* LOF variants appeared to be able to affect the hypertriglyceridemic trait in affected children. Based on these results we could postulate that abnormality in LPL, even though not causative, might favour the development (or the worsening) of the hypertriglyceridemic trait in, at least, a subset of AD children. It is well known that mild-to-moderate hypertriglyceridemia as well as lower HDL-C are typically multigenic [36] and therefore other genes might be responsible of susceptibility to the AD phenotype in the youth. Further resequencing studies in larger cohorts of AD children will be necessary to shedding more light on these aspects.

## Conclusions

Our study demonstrated that AD is a common dyslipidemia in childhood and this definition, although based on rather arbitrary cut-off criteria, identified a group of children showing several underlying metabolic abnormalities, many of which appear to be under a genetic influence. In particular, our data might suggest that the interaction between abnormalities in insulin sensitivity and in the metabolism of apoB-containing lipoprotein could represent early events in AD, which might later associate with unfavorable changes in visceral fat deposition. Moreover, some dyslipidemic children also carry abnormalities in the LPL activity, which may also give contribution to a hypertriglyceridemic component of AD. Overall these findings, not only confirm the complex nature of AD, but also have practical clinical implications. In fact, they strongly suggest that an early detection of children with AD would allow the identification of individuals highly prone to develop cardiometabolic risk factors in adulthood and therefore would most benefit of earlier preventive interventions.

## Supporting Information

S1 File Supplementary Methods. (PDF)

S1 Table Primers for PCR amplification and cycle sequencing of *APOA1* and *LPL* genes. (PDF)

S2 Table Primers for minisequencing of *LPL* gene. The underlined nucleotides are the mobility tail added.
(PDF)

S3 Table Correlations between variables in the whole group of study children (n = 283). (PDF)

S4 Table Heritability (h2) values and effect of covariates (age, sex and their interaction) in the whole sample and stratified for case/control families. (PDF)
