# Correction: Atherogenic Dyslipidemia in Children: Evaluation of Clinical, Biochemical and Genetic Aspects

**DOI:** 10.1371/journal.pone.0133335

**Published:** 2015-07-15

**Authors:** Anna Montali, Gessica Truglio, Francesco Martino, Fabrizio Ceci, Giampiero Ferraguti, Ester Ciociola, Marianna Maranghi, Francesco Gianfagna, Licia Iacoviello, Roberto Strom, Marco Lucarelli, Marcello Arca

There is an error in affiliation 5 for authors Francesco Gianfagna and Licia Iacoviello. Affiliation 5 should be: Department of Epidemiology and Prevention, Laboratory of Molecular and Nutritional Epidemiology, Istituto di Ricovero e Cura a Carattere Scientifico, Istituto Neurologico Mediterraneo Neuromed, Pozzilli, Italy.

## References

[1] Montali A, Truglio G, Martino F, Ceci F, Ferraguti G, Ciociola E, et al (2015) Atherogenic Dyslipidemia in Children: Evaluation of Clinical, Biochemical and Genetic Aspects. PLoS ONE 10(4): e0120099 doi: 10.1371/journal.pone.0120099 25897955 10.1371/journal.pone.0120099 PMC4405441

