# Supplementary material for: Atherogenic Dyslipidemia in Children: Evaluation of Clinical, Biochemical and Genetic Aspects
Source: PLoS One. 2015 Apr 21;10(4):e0120099. doi: 10.1371/journal.pone.0120099 (PMC4405441; doi:10.1371/journal.pone.0120099)
Supplement: S1 File — (PDF) [file pone.0120099.s001.pdf]

Supplementary Methods

Genetic Analysis

The promoter and all 4 exons and introns of APOA1 gene and the promoter and the 10 exons (with adjacent intronic regions) of LPL gene were analyzed. In particular, the APOA1 gene was divided into 4 zones for the PCR amplification (**S2 Table A**); each zone was subsequently divided into two parts for sequencing (**S2 Table B**). For the LPL gene, the primers flanking the exons were used for both PCR amplification and sequencing (**S2 Table C**). PCR amplifications were performed in a total volume of 15 µl with 50 ng of genomic DNA, 6 pmols of each primer, 0.25 units of Go Taq hot start polymerase (Promega, USA), 175 µM of each dNTP (Fermentas, USA), 1.5 mM $MgCl_2$ and 1x manufacturer's buffer. The PCR cycle was the following: 2' 95°C; 35 cycles of 45'' 94°C, 1' 30'' 58°C, 2'30'' 72°C followed by 7' 72°C. PCR products were purified by a common protocol based on thermosensitive alkaline phosphatase (Fermentas, USA) and exonuclease I (USB Corporation, USA). Sequencing reactions were performed by using the Big Dye Terminator Reaction Kit version 1.1 (Applied Biosystems, USA) according to the manufacturer's instructions by using the genetic analyzer ABI PRISM 3130*xl* (Applied Biosystems, USA). Sequencing products were purified by Montage SEQ96 sequencing reaction cleanup kit (Millipore, USA) following manufacturer instructions and subsequently analyzed by the genetic analyzer. The sequence data were analyzed by using the software SeqScape (Applied Biosystems, USA) personalized for the semi-automatic recognition of sequence variations possibly found in the studied zones.

The SNPs identified in the LPL gene were investigated in the general population by the SNaPshot assay (Life technologies, USA). Briefly, the method consisted in the amplification of regions containing SNPs by a multiplex PCR of 5'-UTR and exons 2, 6, 8 and 9 of LPL gene (**S2 Table C**) that was subsequently purified by the exonuclease I – alkaline phosphatase method. Purified PCR products underwent extension reactions by using the primers reported in the **S3 Table** and in the presence of fluorescent ddNTPs. The polymerase extends the primer by only one nucleotide, adding a single ddNTP at its 3' end. After alkaline phosphatase purification, extension products were analyzed by the genetic analyzer. The data analysis was performed by a personalized template for Genotyper software (Applied Biosystems, USA).
