# Supplementary material for: Atherogenic Dyslipidemia in Children: Evaluation of Clinical, Biochemical and Genetic Aspects
Source: PLoS One. 2015 Apr 21;10(4):e0120099. doi: 10.1371/journal.pone.0120099 (PMC4405441; doi:10.1371/journal.pone.0120099)
Supplement: S1 Table — (PDF) [file pone.0120099.s002.pdf]

S2 Table. Primers for PCR amplification and cycle sequencing of *APOA1* and *LPL* genes.

A) Primers for PCR amplification of APOA1.

ANALYZED ZONE	FORWARD	REVERSE	PRODUCT SIZE (bp)	Ta (°C)
5' UTR - exon 3	5'-TCCTCTGCCAACACAATGG-3'	5'GGCCAGGTCCTTCACTCG-3'	878	58
Exon 2 – exon 4	5-TCCTGACGGGTAGGTGTCC-3'	5'-GCTGTCCCAGTTGTCAAGG-3'	970	58
Intron 3 - 3' UTR (up)	5'-CCAGCTCTGTCTCCTTTTTAGC-3'	5'-GCACGGAGTTGTTGAGATCC-3'	981	58
Exon 4 - 3' UTR (down)	5'-GAGCTTCAAGGTCAGCTTCC-3'	5'-TCCTGGCCTGAAGTGATACG-3'	1151	58

B) Primers for cycle sequencing of APOA1.

ApoA1	FORWARD	REVERSE	PRODUCT SIZE (bp)	Ta (°C)
5' UTR (up) – intron 1	5'-CTCCCATGGAGGGGAAGG-3'	5'-CACAGAGCGGGAGAAGACC-3'	421	58
5' UTR (down) – intron 2 (down)	5'-TGGCTGCAGACATAAATAGGC-3'	5'-CTGAGATCTGAGCCGAAAGG-3'	465	58
Intron 2 (up) – intron 3 (up 1)	5'-CCTAACCTAGGGAGCCAACC-3'	5'-GCCCAGCTCATCAGATATTAGG-3'	441	58
Exon 3 – intron 3 (down)	5'-GCGGCAGAGACTATGTGTCC-3'	5'-GCAGTGGCCTAGCATTTCC-3'	554	58
Intron 3 (up 2) – exon 4 (down)	5'-CCTTTTTAGCTCCTCTCTGTGC-3'	5'-AGCTCGTGCAGCTTCTGG-3'	476	58
Exon 4 (up) – 3' UTR (up)	5'-AGCTCTACCGCCAGAAGG-3'	5'-AAGCTGCTTCCCACTTTGG -3'	459	58
Exon 4 (down) – 3' UTR (down)	5'-AAGCTCAACACCCAGTGAGG-3'	5'-TAGTGGGAGGGGGATACTGG-3'	657	58
3' UTR (distal)	5'-GAAGCGCTTGGAGTTTGC-3'	5'-TGATACGCCCACCTCAGC-3'	537	58

C) Primers for PCR amplification and cycle sequencing of LPL.

ANALYZED ZONE	FORWARD	REVERSE	PRODUCT SIZE (bp)	Ta (°C)
5' UTR	5'-CGTGAATCGATGTAAACCTGTG-3'	5'-GTTTGGCGCTGAGCAAGT-3'	377	58
Exon 1 and adjacent intronic zones	5'-CAGTCACATAAGCAGCCTT-3'	5'-TTCCAACTTCCTTCTTCTC-3'	401	58
Exon 2 and adjacent intronic zones	5'-CGTTGGAGCATCTGTTGTTCT-3'	5'-ATGTGGGGTGCGGACCCATC-3'	427	58
Exon 3 and adjacent intronic zones	5'-TTGGGCTGATGTATCTATGAC-3'	5'-GGCAGGACTATTTATTGGC-3'	451	58
Exon 4 and adjacent intronic zones	5'-ATTTTTGGCAGAACTGTAAGCAC-3'	5'-AAATCTGCAGGCATTGTGTG-3'	408	58
Exon 5 and adjacent intronic zones	5'-TCATTTTAGAAGGAGCCAAG-3'	5'-GTGGTCATGCTGAACATTG-3'	569	58
Exon 6 and adjacent intronic zones	5'-ACTCTCTTGAAGGTGGGTGG-3'	5'-ATGGGAATTAGAAGCCTCAGAC-3'	624	58
Exon 7 and adjacent intronic zones	5'-TTCCATGTGTGTGCACTTCC-3'	5'-CTTGTTTTCTAGGCATCGCTCT-3'	350	58
Exon 8 and adjacent intronic zones	5'-GCAGGGAGAGCTGATCTCTATAA-3'	5'-TGTGAAGGCCCCTGAAATAC-3'	318	58
Exon 9 and adjacent intronic zones	5'-CCCTCTGATTCTGATGTGGC-3'	5'-CTGGTGATGGGGTGAAGAG-3'	363	58
Exon 10 and intron 9 adjacent intronic zone - 3' UTR	5'-GTCTTCCATTCCTACAAACCTG-3'	5'-TTTTACTCTGCGCTCAAACAAT-3'	497	58
