# Supplementary material for: Atherogenic Dyslipidemia in Children: Evaluation of Clinical, Biochemical and Genetic Aspects
Source: PLoS One. 2015 Apr 21;10(4):e0120099. doi: 10.1371/journal.pone.0120099 (PMC4405441; doi:10.1371/journal.pone.0120099)
Supplement: S2 Table — The underlined nucleotides are the mobility tail added. (PDF) [file pone.0120099.s003.pdf]

S3 Table . Primers for minisequencing in the LPL gene. The underlined nucleotides are the mobility tail added.

VARIATION	PRIMER
p.Asp9Asn	5’-GGAAAAGAGAAAGGAAAAAAAATTCCAGAAAGAAGAGATTTTATC-3’
p.Ser45Asn	5’-AAAAAACTACCTGTCATTTCAATCACA-3’
p.Asn291Ser	5’-AATCTGGGCTATGAGATCA-3’
p.Leu365Val	5’-AAAGGAAAACACAAATAAGACCTACTCCTTC-3’
p.Ser447*	5’-AGAAAAAAGGGAAAAATGACAAGTCTCTGAATAAGAAGT-3’
