# Supplementary material for: Atherogenic Dyslipidemia in Children: Evaluation of Clinical, Biochemical and Genetic Aspects
Source: PLoS One. 2015 Apr 21;10(4):e0120099. doi: 10.1371/journal.pone.0120099 (PMC4405441; doi:10.1371/journal.pone.0120099)
Supplement: S3 Table — (PDF) [file pone.0120099.s004.pdf]

S4 Table. Correlations between variables in the whole group of study children (n=283)

Variables	Age	TC	HDL-C	TG	LDL-C	FBG	Insulin	hs-CRP	Apo B	Apo A1	Adiponectin	HOMA-IR	BMI
**TC**	0.025												
**HDL-C**	-0.006	0.224**											
**TG**	0.070	0.008	-0.532**										
**LDL-C**	0.009	0.944**	-0.013	-0.096									
**FBG**	0.221**	0.010	-0.224**	0.300**	0.008								
**Insulin**	0.486**	-0.101	-0.312**	0.356**	-0.089	0.246**							
**hs-CRP**	-0.009	-0.082	-0.154*	0.007	-0.021	-0.055	0.160*						
**ApoB**	0.020	0.579**	-0.144*	-0.014	0.671**	0.046	0.016	0.040					
**Apo A1**	0.070	0.130*	0.589**	-0.267**	-0.024	-0.096	-0.166*	-0.138*	-0.042				
**Adiponectin**	-0.381**	0.141	0.224*	-0.142	0.093	-0.061	-0.353**	-0.113	0.023	0.054			
**HOMA-IR**	0.483**	-0.066	-0.332**	0.420**	-0.063	0.424**	0.970**	0.151*	0.043	-0.146*	-0.343**		
**BMI**	0.410**	-0.006	-0.275**	0.323**	0.010	0.340**	0.575**	0.043	0.076	-0.163*	-0.413**	0.587**	
**Waist circ.**	0.617**	-0.060	-0.373**	0.467**	-0.044	0.190	0.712**	0.151	0.066	-0.153	-0.319*	0.649**	0.841**

TC, total cholesterol; HDL-C, high density lipoprotein cholesterol; TG, triglycerides; LDL-C, low density lipoprotein cholesterol; FBG, fasting blood glucose; hs-CRP, high sensitivity C Reactive Protein; HOMA-IR, HOmeostasis Model Assessment of IR index; BMI, body mass index

*p< 0.05; **p< 0.01
