# Supplementary material for: Atherogenic Dyslipidemia in Children: Evaluation of Clinical, Biochemical and Genetic Aspects
Source: PLoS One. 2015 Apr 21;10(4):e0120099. doi: 10.1371/journal.pone.0120099 (PMC4405441; doi:10.1371/journal.pone.0120099)
Supplement: S4 Table — (PDF) [file pone.0120099.s005.pdf]

S5 Table. Heritability (h2) values and effect of covariates (age, sex and their interaction) in the whole sample and stratified for case/control families.

	Overall (1052 subjects, 283 families)			AD families (229subjects, 62 families)			Control families (823subjects, 221families)		
	**h2 (SE) (%)**	***P* values**	**Covariates (%)**	**h2 (SE) (%)**	**P values**	**Covariates (%)**	**h2 (SE) (%)**	**P values**	**Covariates (%)**
BMI	**44.1 (5.7)**	9.E-15	**53.7**	**52.1 (11.4)**	6.E-06	**33.5**	**40.8 (6.6)**	2.E-10	**57.9**
Waist circumference	**29.7 (9.9)**	1.E-03	**67.6**	**45.6 (17.8)**	6.E-03	**55.0**	**17.7 (11.9)**	0.06	**80.1**
Hip circumference	**42.2 (12.4)**	4.E-04	**0.2**	**78.9 (18.0)**	1.E-04	**60.6**	**22.4 (15.9)**	0.08	**0.0**
PAD	**15.8 (8.2)**	2.E-02	**24.4**	**Ne**	-		**22.5 (9.1)**	5.E-03	**24.4**
PAS	**18.1 (8.8)**	2.E-02	**30.1**	**5.3 (21.2)**	0.40	**28.5**	**20.4 (9.6)**	1.E-02	**30.9**
Glucose	**88.9 (3.7)**	4.E-59	**17.1**	**90.4 (7.8)**	8.E-15	**13.9**	**86.2 (4.4)**	1.E-42	**18.5**
Insulin	**22.1 (6.4)**	1.E-04	**2.6**	**26.9 (14.3)**	3.E-02	**0.2**	**18.9 (7.2)**	3.E-03	**6.4**
HOMA	**32.2 (6.8)**	5.E-07	**3.0**	**30.9 (15.8)**	2.E-02	**0.0**	**26.8 (7.7)**	2.E-04	**7.2**
Total cholesterol	**45.7 (6.1)**	7.E-14	**15.0**	**71.2 (10.8)**	2.E-08	**20.5**	**37.5 (7.1)**	3.E-08	**14.3**
HDL	**63.5 (4.6)**	1.E-35	**13.5**	**32.8 (13.5)**	5.E-03	**21.5**	**48.3 (6.1)**	5.E-15	**18.8**
Non-HDL	**55.2 (5.5)**	3.E-21	**12.7**	**68.6 (11.5)**	4.E-07	**8.7**	**48.7 (6.3)**	2.E-14	**14.9**
Non-HDL/HDL ratio	**66.2 (4.9)**	2.E-31	**22.3**	**47.4 (14.8)**	1.E-03	**10.7**	**55.0 (6.2)**	1.E-16	**29.4**
LDL	**58.9 (5.4)**	6.E-25	**8.4**	**74.6 (9.5)**	2.E-10	**8.8**	**52.5 (6.3)**	2.E-16	**8.8**
Tryglicerides	**58.4 (5.6)**	3.E-22	**27.3**	**28.7 (13.7)**	1.E-02	**5.9**	**45.0 (6.7)**	4.E-11	**28.2**
ApoAI	**67.0 (4.7)**	2.E-35	**8.1**	**51.4 (10.7)**	2.E-06	**12.5**	**66.4 (5.7)**	3.E-25	**8.3**
ApoB	**74.8 (4.8)**	6.E-36	**13.5**	**81.3 (9.9)**	8.E-10	**11.2**	**73.0 (5.4)**	9.E-29	**13.6**
ApoAI/HDL ratio	**72.2 (5.1)**	3.E-33	**13.5**	**57.8 (12.9)**	2.E-05	**12.2**	**64.7 (6.4)**	1.E-19	**19.3**
ApoB/non-HDL ratio	**83.0 (4.1)**	1.E-51	**0.0**	**77.3 (9.6)**	4.E-10	**0.2**	**84.5 (4.5)**	1.E-42	**0.7**
ApoB/Apo-aI ratio	**75.8 (4.8)**	6.E-38	**11.6**	**60.8 (11.5)**	1.E-06	**6.4**	**76.9 (5.3)**	2.E-31	**12.9**
Adiponectin	**16.8 (13.2)**	0.10	**15.5**	**7.6 (17.3)**	0.33	**7.2**	**43.8 (18.7)**	2.E-02	**22.0**
CRP	**23.4 (7.4)**	5.E-04	**8.4**	**20.7 (14.4)**	0.07	**0.0**	**25.2 (8.6)**	1.E-03	**14.8**
MetSyn	**65.5 (16.9)**	8.E-05	**1.82***	**Ne**	-		**49.6 (147.2)**	0.40	**20.5***

*****Kullback-Leibler R-squared for categorical variables
